# Incense Burning Indoor Pollution: Impact on the prevalence of prediabetes and Type-2 Diabetes Mellitus

**DOI:** 10.12669/pjms.38.7.6189

**Published:** 2022

**Authors:** Thamir M. Al-khlaiwi, Sultan Ayoub Meo, Syed Shahid Habib, Imran Muhammad Umar Meo, Mohammed S. Alqhtani

**Affiliations:** 1 Thamir M. Al-khlaiwi, Department of Physiology, College of Medicine, King Saud University, Riyadh, Saudi Arabia; 2 Sultan Ayoub Meo, Department of Physiology, College of Medicine, King Saud University, Riyadh, Saudi Arabia; 3 Syed Shahid Habib, Department of Physiology, College of Medicine, King Saud University, Riyadh, Saudi Arabia; 4 Imran Muhammad Umar Meo, Department of Physiology, College of Medicine, King Saud University, Riyadh, Saudi Arabia; 5 Mohammed S. Alqhtani, Department of Physiology, College of Medicine, King Saud University, Riyadh, Saudi Arabia

**Keywords:** Incense pollution, Occupational hazards, Prevalence, Type2 diabetes mellitus

## Abstract

**Objectives:**

Incense burning is a well-known practice in Asian and Middle Eastern cultures for ceremonial and religious purposes. The excessive use of incense burning has become a critical environmental health concern. The incense sellers are more exposed to incense allied air pollution. This study examines the association between prediabetes and type 2 diabetes mellitus (T2DM) in incense sellers.

**Methods:**

This cross-sectional prevalence study was conducted in the Department of Physiology, College of Medicine, King Saud University, Riyadh, Saudi Arabia” during the period July 2019 to January 2020. After medical history and examinations had been performed, a total of 265 non-smoking volunteers male incense sellers were selected. American Diabetes Association (ADA) criteria were followed, people with “HbA1c less than 5.7% were considered normal; HbA1c 5.7%-6.4% were pre-diabetics, and HbA1c > 6.4% were considered people with diabetes”. In shops, the incense sellers were exposed to incense-related pollution for 8 hours daily, seven days a week. The mean age for the participants was 25±5.5 years, and body mass index was 19±2.8 (kg/m)^2^.

**Results:**

In incense sellers, the pre-diabetic was 125 (47.2%), and diabetes was 75 (28.3%). However, 65 (24.5 %) incense sellers were without prediabetes and diabetes. There was an increase in HbA1c levels with increasing working exposure to incense shops.

**Conclusions:**

The prevalence of pre-diabetic and type-2 diabetes was increased in incense sellers. The prevalence of pre-diabetic and type-2 diabetes was further increased with the increasing working duration of incense sellers. The study findings call for safe practice and avoiding indoor burning incense. It is suggested that well-ventilated areas with proper masks for the workers may reduce the incense-related pollution effects.

## INTRODUCTION

The use of incense is a prominently essential part of Asian and Middle Eastern cultures. Burning incense is recognized as one of the significant constituents of indoor air pollution. Consequently, many human diseases are suspected to be linked to incense burning.^1^ In general, burning incense releases smoke containing hazardous substances to jeopardize human health. The burning of incense harms human health that mimics tobacco’s health risk. The constituents of incense smoke are very similar to the compositions of tobacco smoke. Incense smoke contains mainly mixtures of ultrafine and fine particles that can be inhaled, are inherently hypertoxic, and contribute to many health issues.^1^ Some of these identified particles are Auramine O (AuO), particulate matter (PM), “polycyclic aromatic hydrocarbons (PAHs), pyrene, and volatile organic compounds (VOCs) such as benzene” were detected along with hazardous gases.^2^ The cytotoxicity of PM particles was evaluated in cell biology, and the viabilities of cells were significantly compromised. The cellular damage response, inflammation, and oxidative stress were well reported in the literature.^3^

Auramine O (AuO) is a chemical that could have a carcinogenic effect on human health. The literature highlights that the accumulation of AuO promote the lung cancer and enhanced invasive abilities in vitro and vivo.^2^ The literature also demonstrates that incense particles, including AuO can enhance cellular proliferation and non-small cell lung cancer.^4^

The literature also demonstrated the correlation between burning incense and pulmonary function impairment in children and adolescents. The study findings showed that burning incense was associated with respiratory symptoms, including allergic rhinitis, dry cough, phlegm production, bronchitis, and bronchiolitis.^5^ Moreover, exposure to incense smoke can cause respiratory,^6^ cardiovascular diseases,^7^ and abnormal kidney functions.^8^

The effects of several environmental pollutants on the occurrence of prediabetes and diabetes mellitus have been examined and showed a high association.^9^ Industrial workers worldwide are working daily in various occupational environments and are subsequently exposed to multiple pollution manifestations such as oil, wood, flour, welding, cement, and plastic industry and becoming more prone to diabetes.^10^ However, there is no available literature regarding incense sellers directly affected by incense-related pollution. This novel study examined the association between prediabetes and type 2 diabetes mellitus (T2DM) in incense sellers.

## METHODS

This “cross-sectional prevalence study was conducted in the Department of Physiology, College of Medicine, King Saud University, Riyadh, Saudi Arabia” during the period July 2019 to January 2020. After history and examination, a total of 265 non-smoking volunteers male incense sellers were selected. The incense sellers had been exposed to incense-related pollution for eight hours daily, seven days a week. American Diabetes Association (ADA) criteria were followed, people with “HbA1c less than 5.7% were normal; HbA1c 5.7%-6.4% were pre-diabetics”, and HbA1c > 6.4% were considered people with diabetes. All participants were given written consent to join the research project. After reading the research objectives, the participants choose to continue or withdraw from the research project.

### Exclusion Criteria

Subjects with a history of known diabetes mellitus, blood diseases such as anemia and blood transfusion, and malignancy were excluded from the study. Incense sellers who smoke cigarettes or shisha were excluded to minimize the impact of smoking on the findings. Any incense seller who had a history of working in cement, wood, oil, welding, and flour plants was excluded from the study.

Clinical characteristics and history were obtained by two co-investigators interviewing 265 volunteer incense sellers. All variables were acquired, including age, weight, BMI, and duration of exposure to incense-related pollution.

### Measurements of Glycated Hemoglobin

The HbA1c was determined using the “Clover A1c system (Inforpia, Kyunggi, Korea), an automated boronate affinity assay to determine the HbA1c percentage in whole blood”. American Diabetes Association (ADA) criteria were followed with “HbA1c less than 5.7% were considered normal; HbA1c 5.7%–6.4% were pre-diabetics, and HbA1c > 6.4% were considered people with diabetes”.^11^ The American Diabetes Association has recommended HbA1c as one of the standard indicators for a reproducible glycemic history of the previous 3–4 months.

### Statistical Analysis

The “SPSS version 22 and Microsoft Windows” were used for statistical analysis. All the variables were “expressed as the mean ± standard deviation, and descriptive data were expressed as percentages (%). The percentages for the prevalence of prediabetes and diabetes, their association with age, BMI, and duration of incense exposure were calculated”. Spearman’s correlations were determined between HbA1c and other variables. Linear by linear association was calculated from the Chi-square test between years of exposure categories and HbA1c levels. The significance level was presumed at p < 0.05.

### Ethics Approval

The study protocol was approved by the “Institutional Review Board, Ethics Committee, College of Medicine Research Centre, King Saud University (Ref: E-18-3654/ 2019)”.

## RESULTS

**Fig.1:**
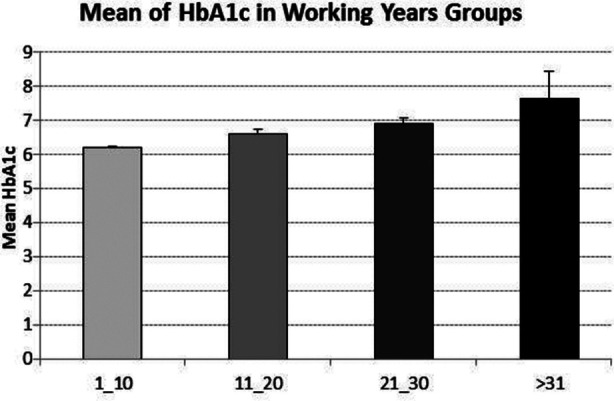
Association with HbA1c with duration to exposure to incense.

The anthropometric characteristics of the incense sellers are shown in Table-I. All participants’ mean age was 25±5.5 years; BMI 19±2.8 (kg/m)2; and duration of working exposure in the incense shop was 6.7 years. The prevalence of prediabetes and T2DM in non-smokers incense sellers involved in incense selling is shown in Table-II. Based on HbA1c and ADA classification, all the “participants were divided into three groups: non-diabetics HbA1c less than 5.7%; pre-diabetics HbA1c 5.7%–6.4%; and diabetics HbA1c >6.4%”. In incense sellers, the prevalence of prediabetes was 125 (47.2%), and T2DM was 75 (28.3%). However, 65 (24.5 %) incense sellers’ HbA1c was within the normal range. Fig.1 and Table-III show a significant increase in the prevalence of prediabetes and T2DM among incense sellers, increasing working exposure (duration of exposure to incense with p value= .05). There was a significant positive correlation between years of exposure and HbA1c levels (P<0.001, r=.713^**^) (Table-III)

**Table I:** Study participant characteristics (n=265).

Variable	Mean (SD)
Age (years)	25±5.5
BMI (kg/m2)	19±2.8
Years of exposure	6.7

***Note:*** Values are expressed in mean and standard deviation.

**Table II:** Number and percentage of different categories of incense sellers according to glycemic states (n=265).

Parameters	Number(%)
Normal	65(24.5 %)
Prediabetic	125 (47.2%)
Diabetic	75 (28.3%)
Prediabetic and diabetic	200 (75.5%)

**Table III:** Prevalence of prediabetes and T2DM in incense workers (n=265).

Variables	Nondiabetics (n=65)	Pre diabetics (n=125)	Diabetics (n=75)	F-value	p-value
Parameters	HbA1c <5.7%	HbA1c 5.7%-6.4%	HbA1c >6.4%		
Age (years)	31 ±9.69	34.032 ± 8.849	40.558± 9.93	24.122	0.0001
BMI	27.120 ± 5.1432	26.46 ± 5.194	29.03±5.11	10.525	0.0001
Exposure (ys)	3.09 ±7.907	8.51 ±6.969	16.285±8.048	76.160	0.0001

***Note:***"Values are expressed in mean ± SD. HbA1c values are presented based on American Diabetic Association. Guidelines 2018. BMI = Body Mass Index; SD = standard deviation; HbA1c = glycated hemoglobin".

**Table IV:** Spearman’s Correlations between HbA1c and different variables.

Parameters	Age	Weight	Height	BMI	Working years	HbA1c
Age	1.000	.125*	-.090	.179**	.580**	.437**
Weight	.125*	1.000	.513**	.834**	.135*	.144*
Height	-.090	.513**	1.000	.047	-.033	-.029
BMI	.179**	.834**	.047	1.000	.166**	.209**
Working (ys)	.580**	.135*	-.033	.166**	1.000	.713**
HbA1c	.437**	.144*	-.029	.209**	.713**	1.000

* . Correlation is significant at the 0.05 level (2-tailed).

** . Correlation is significant at the 0.01 level (2-tailed). Linear by linear Chi-square association p-value <0.001

## DISCUSSION

This is the first novel study that examines the correlation between prediabetes and T2DM in incense sellers who are closely affected by incense-related pollution. This study identified that prediabetes and diabetes mellitus were increased in the incense sellers in general and increased with more exposure to incense.

Incense burning causes air pollution and generates many hazardous pollutants including, “particulate matter (PM), carbon monoxide (CO), carbon dioxide (CO2), sulfur dioxide (SO2), nitrogen dioxide (NO2), volatile organic compounds, aldehydes and polycyclic aromatic hydrocarbons (PAHs)” are released into the air. Incense burning is considered a primary source of air pollution, mainly in shops, schools, homes, and indoor cultural activities. The literature also acknowledges that incense burning is a leading cause of indoor air pollution.^12^ Worldwide, approximately 3.2 billion people globally are exposed to incense-burning air pollutants with the widespread use of incense as worshiping rituals across different cultures in various contents.^12^ Air pollution has become one of the factors that can increase the prevalence of T2DM.^13^,^14^

More recently, worldwide literature highlights the linkage between various air pollutants and the rising risk of diabetes mellitus. Wang et al., 2021^15^, Li et al.,^16^, and Lee et al., 2021^17^ reported a positive and strong relationship between long-standing exposure to air pollutants, “PM2.5, Ozone (O3), SO2” with a high-risk T2DM. Moreover, the magnitude of association became more robust as the duration of exposure increased. Similarly, in the present study, prediabetes and diabetes mellitus have been observed in the incense sellers and correlated with the duration of exposure. (Table-III).

Incense-related pollutants could be part of oxidative stress,^18^ inflammation, endocrine-disrupting, insulin signaling, glucose metabolism impairment, and insulin resistance.^19^,^20^ These possible mechanisms for the adverse effect of air pollution can participate in insulin resistance and subsequently in prediabetes and diabetes mellitus. Comprehensive research is required to understand how incense-related pollution can affect predicates and T2DM.

### Study Strengths and Limitations

This is the first study to examine the correlation between prediabetes and type-2 diabetic mellitus in incense sellers. The study exclusion criteria were highly standardized, and the “American Diabetes Association classification and diagnosis approach” was employed. The limitations of this study are: that it is a cross-sectional study. Some workers refused to participate because of their employment issues despite the free test and the confidentiality of personal identities. In addition, difficulties in finding non-smoking incense sellers to eliminate the effect of smoking. Further studies with a larger sample size and the analysis of different incense-related pollutants in the human body are of value to understanding the exact mechanism and current evidence of the role of incense-related pollution and the occurrence of prediabetes and diabetes mellitus among incense workers.

## CONCLUSIONS

Exposure to incense-related pollution causes an increasing occurrence of prediabetes and type 2 diabetes mellitus among incense sellers. The incense sellers should wear a protective face mask to minimize the risk of pollutants exposure. In addition, a well-ventilated area is required to reduce the effects of incense pollution. Public awareness should be considered to educate people about the side effects of incense burning. The decision-makers in health authorities should take advanced measures to reduce incense-related pollution and subsequently reduce the occurrence of prediabetes and T2DM.

### Authors’ Contribution:

**TA, SAM** conceived, designed, wrote & edited the manuscript.

**SS, IM** statistical analysis.

**MA** data collection.

**TA** is responsible and accountable for the accuracy and integrity of the work.
